# The Relationship between Gentle Tactile Stimulation on the Fetus and Its Temperament 3 Months after Birth

**DOI:** 10.1155/2015/371906

**Published:** 2015-05-28

**Authors:** Zhe-Wei Wang, Jing Hua, Yu-Hong Xu

**Affiliations:** ^1^Department of Obstetrics and Gynaecology, Shanghai First Maternity and Infant Hospital, Tongji University, 536 Changle Road, Shanghai 200040, China; ^2^Department of MCH, Shanghai First Maternity and Infant Hospital, Tongji University, 536 Changle Road, Shanghai 200040, China

## Abstract

*Objective.* The aim of this study was to evaluate the effect of gentle tactile stimulation on the fetus in its temperament 3 months after birth.* Method.* A total of 302 mother-3-month-infant dyads enrolled the retrospective cohort study. 76 mothers had regular gentle tactile stimulation on the fetus in their pregnancy; 62 mothers had irregular tactile stimulation on the fetus, and the rest of 164 mothers who had no tactile stimulation served as nonexposure group. Temperament was assessed using the EITS (a nine-dimensional scale of temperament). *Results.* Significant difference in temperament type was found among infants in 3 groups at 3 months of age. In the regular practice group, the babies with easy type temperament accounted for 73.7%, which was higher than that in irregular practice group (53.2%, *P* = 0.012) and that in the control group (42.1%, *P* < 0.001). Compared to infants in no practice group, the infants who had received regular gentle tactile stimulation before birth were lower in negative mood (*P* = 0.047) while higher in adaptability (*P* < 0.001), approach (*P* = 0.001), and persistence (*P* = 0.001), respectively. *Conclusion.* Regular gentle tactile stimulation on fetus may promote the formation of easy type infant temperament.

## 1. Introduction

Temperament refers to individual differences in an infant's expressions of arousal and emotion and describes infant self-regulation, reactivity, and modulation [1]. It was initially defined as stable, biologically based individual differences in reactivity and regulation that form the core of personality [2], but recently it has been viewed as a predisposing set of individual characteristics with the potential to systematically change over time as a child develops [3, 4]. Infant temperament has been classified into 4 types: easy, intermediate, difficult, and slow to warm up. According to the measure standard, the infant with easy type temperament is more amiable, regular in living, and open than the infant with the other temperaments. The difficult type infant is more active, irritable, and irregular in living. The slow type baby is slower in response to environment, is more socially withdrawn, and has less interest in the people around.

Fetus has sense and feeling in uterus. Neuronal multiplication occurs mainly from the 10th to the 20th gestational weeks. Somatosensory functions are developed at an early stage [5]. While the basic physical structure of the sensory receptors develops early in gestation, most of the neurosensory development occurs in the last sixteen to twenty weeks [6]. The human fetus is able to feel pain after 24-week gestation [7]. During the seventh month, the fetus begins to show signs of personality and intentional behavior [8]. Some findings provide evidence of fetal attention and memory after 32 gestational weeks [9]. The fetus has its psychology: feeling, dreaming, and even enjoying [10]. Babies are particularly sensitive to touch, which is the most highly developed sense at birth [11].

Since the 1960s, there have been numerous studies that have shown the benefits of massage on the development of newborns and children referring to dimension of physics and psychology [12–25]. In addition, preterm neonates who received massage showed more mature habituation and orientation [26], better results on developmental tests [27], and fewer stress behaviors [28] than control infants. All these affected psychological elements are all related to temperament. Massage therapy by mothers combined with skin-to-skin care during neonatal hospital stay could improve child's neurodevelopment outcome at 2-year corrected age [29]. Infant's temperament is influenced by the mother's personality, family economic status, pregnancy-specific anxiety, depression and stress in pregnancy and after delivery, parenting, and mode of feeding [4, 30–34]. Prenatal attachment has been discussed in the literature for decades and has evolved in recent years into the construct of maternal-fetal attachment (MFA). Cranley developed the Maternal-Fetal Attachment Scale (MFAS) to measure the construct [35]. Music fetal education means playing music to fetal, which is also one kind of maternal fetal attachment as talking to the fetus and tactile stimulation on the fetus. Nowadays, gentle abdominal tactile stimulation through abdomen in pregnancy is one way of maternal-fetal attachment. Some study has testified that massage can relieve the pregnant anxiety [36]. Although there is considerable volume of international research on infant massage from the scope of physics and psychology, surprisingly little scientific research has been conducted on the topic of gentle tactile stimulation on the fetus. We speculated that when the pregnant women give gentle tactile stimulation on the fetus, she may feel fetus' response more and her abdomen also receives more massage than often. These may relieve her anxiety and depression and reduce the level of cortisol in blood and amniotic fluid, which is testified to have indirect effects on infant temperament [37]. Meanwhile we hypothesized that tactile stimulation through abdomen promotes the flow of amniotic fluid and enhances the gentle stimulation on the fetal skin and may has not only physical effect, but also psychological effect similar to that of massage on preterm babies.

The maternal-fetal attachment education center in our hospital has been opened for about 20 years and there are about 150 pregnant women who receive the education through the lecture in every month. So we could have enough samples for study on maternal-fetal attachment. In the present study, we have tested the hypothesis that regular gentle tactile stimulation contributes to the formation of easy type infant temperament 3 months after birth. The result is hoped to find the availably effective method for the easy type infant temperament in the future.

## 2. Subjects and Methods

### 2.1. Subjects

A total of 302 mother-3-month-infant dyads enrolled the retrospective cohort study by sequential sampling, using the registration number in August 2014. The inclusion criteria were mothers willing to participate. The exclusion criteria were smoking or drinking during pregnancy, having serious mental illness, having obstetric complications such as maternal organ diseases, obstetric-special diseases, or fetal abnormality, premature delivery, prenatal mortality, or newborn abnormalities (Apgar score < 8, birth weight < 2500 g, malformation, or other problems).

### 2.2. Study Design

#### 2.2.1. Exposure Factor and Groups

The retrospective study was designed among the 3-month postpartum women who attended the public health lecture in our hospital. Exposure factor is gentle abdominal tactile stimulation on the fetus during pregnancy. The method was based on “Modern maternal-fetal attachment” which was edited by Jiangdixian and published in January 2014 by Second Military Medical University press. The method was described on pages 48–63. The theory and method of musical fetal education, gentle tactile stimulation on the fetus, and talking to fetus were transferred by lessons. The key features of this method were (1) duration: performing from the 24th gestational week until delivery; (2) interval: performing for 5 minutes, once or twice a day, on a fixed time schedule; (3) maneuver: the activities including moderate shaking, striking on the abdomen, and mild pressing of fingers into the abdomen where fetal body can be felt; (4) Stopping immediately: when feeling any abdomen pain, bleeding, or fetal movement becoming fierce. Mothers did not continue with massage postnatally. Engaging in these practices, in accordance with all 4 points, was defined as regular practice; doing otherwise was considered to be irregular practice. According to this, the study participants were divided into three groups: no practice group (*n* = 164), the irregular practice group (*n* = 62), and the regular practice group (*n* = 76).

#### 2.2.2. Retrospective Data Collection

The postpartum women completed a questionnaire containing data about demographic characteristic, the practice of abdominal tactile stimulation in pregnancy, method of delivery, the newborn's health, and method of feeding. Data were also collected on the regularity of other maternal-fetal attachment ways such as musical fetal education (playing music to fetus) and language fetal education (talking to fetus) as defined in “Modern maternal-fetal attachment.”

#### 2.2.3. Measures

We used 4 psychological scales: Self-Rating Anxiety Scale (SAS, a self-rating scale to measure the depression in the past week whose correlation coefficient with HAMA scale is 0.365), Self-Rating Depression Scale (SDS, a self-rating scale to measure the depression in the past week that has high and moderate correlation with other depression scales such as BDI and HRSD and was translated to Chinese version in 1985), the Eysenck Personality Questionnaire for adults (EPQA, a scale developed by H. J. Eyscenck and B. G. Eysenck in 1952 for personality measurement that has been found to have high reliability and validity, China has revised the version and formulated *T* score tables on the norms of samples in 6 areas and the Chinese research in 30 provinces showed that the reliability and validity of all subscales all meet the requirement of psychological measurement) for mothers, and the Early Infancy Temperament Questionnaire (EITQ, norm scale in Shanghai, China) [38] for 3 month-old infants. The scale is normal in Shanghai, China. Education was final education; income, maternal age, mode of delivery, and feeding method consulted the patients' dictate; maternal temperament consulted EPQA; maternal depression and anxiety after delivery consulted SAS and SDS.

Both SDS and SAS contain 20 items, which are widely used to evaluate the feelings of depression and anxiety. EPQA includes 4 subscales: Extraversion (E), Neuroticism (N), Psychoticism (P), and Lie (L) scale. The Chinese revised version contains 88 items. EITQ, designed by Carey and revised by Chinese experts, assesses the type of temperament of 1–4-month-old infants, according to their primary caregiver [38]. Its internal consistency is between 0.43 and 0.76. The reliability for 3-4-month-old baby is 0.74. The scale consists of 9 dimensions: activity (the frequency of activity and the pace of action in the whole day), rhythmicity (the regularity of Physiological activity such as sleeping and eating), approach/withdrawal (the attitude of accepting or rejecting to the new stimulation), adaptability (the easy degree to adapt himself to the new environment), intensity (the reaction intensity to stimulation), mood (the proportion of pleasant or unpleasant, kind or unkind, happy or unhappy emotion in the whole day), persistence (the tendency to continue to uphold the original activity when encountering setback or difficulties), distractibility (easy degree to shift its attention), and reaction threshold (the stimulation amount needed to cause irritation reaction). The items are rated on a 6-point scale. The higher dimension score indicates higher activity, weaker rhythmicity, lower approach, weaker adaptability, stronger intensity, negative mood, shorter persistence, higher distractibility, and lower threshold.

#### 2.2.4. Target Variable/Outcome

The target variable was the temperament type of the infant, which was measured using EITS, and the difference in infant temperament among the three groups. Multivariate logistic regression was conducted with infant temperament as the dependent variable (1, easy temperament; 0, other infant temperament), with gentle tactile stimulation (0, no practice; 1, irregular practice; 2, regular practice) as the chief independent variable, and using characteristics which were distributed unevenly among the groups as the independent variables: musical fetal education (the method was also based on “Modern maternal-fetal attachment”): 0, no; 1, irregular practice; 2, regular practice; temperament tendency of woman: 1, typical extrovert; 2, ambivert; 3, typical introvert; pregnant woman being only child: 0, no; 1, yes; and annual family income: 1, ≤ 5 000$; 2, 5 000–8 000$; 3, 8 000–16 000$; 4, 16 000–32 000$; 5, ≥32 000$.

### 2.3. Data Analysis

The software named “Cary's children temperament questionnaire testing system” developed by the Xin hua Hospital, Jiaotong University, was used to assess the temperament of the infants. Data analysis was conducted with SPSS 17.0 to compare differences among groups, using chi-square test, Fisher's exact probability test, the Mann-Whitney *U* test, ANOVA test, and multivariate analyses. For all the tests, the 95% confidence was used and a *P* value of 0.05 was considered to be statistically significant.

## 3. Results

### 3.1. Characteristics of the Groups

As shown in Tables 1 and 2, no significant difference was found among groups in terms of age (*F* = 1.353, *P* = 0.260), maternal nerve stability, mother's education, working status, mode of feeding, delivery method, talking to the fetus, and depression and anxiety after delivery. However, statistically significant differences were found in pregnant women's temperament tendency, being only child, musical fetal education, and family income, respectively (*P* < 0.05). And these factors were included in the multivariable analysis as confounding factors.

### 3.2. The Effect of Gentle Tactile Stimulation on the Fetus on Its Temperament 3 Months after Birth

There were notable differences in the types of infant temperament among the three groups (Fisher's exact < 0.001, Table 3). Segmentation of chi-square test indicated that a large majority (73.7%) of the infants in the regular practice group had an easy temperament, more than that (53.2%) in irregular groups (*χ* ^2^ = 6.241, *P* = 0.012) and that (42.1%) in no practice group (*χ* ^2^ = 20.794, *P* < 0.001). However, there was no difference in the ratio of easy type between no practice group and irregular practice group (*χ* ^2^ = 2.260, *P* = 0.133). Although the ratio of difficult type in irregular practice group was higher than the other two groups, there was no significant difference among 3 groups (Fisher's exact = 0.289).  Table 4 showed that gentle regular tactile stimulation was still positively associated with an easy temperament (*P* < 0.001) including other relative factors. As shown in Table 5, ANOVA test showed differences among the three groups on three of the temperament dimensions. Infants who were given regularly gentle fetal tactile stimulation before birth were higher in adaptability (*F* = 12.408, *P* < 0.001), approachability (*F* = 6.064, *P* = 0.003), and persistence (*F* = 5.984, *P* = 0.003). Comparison in pair of groups showed that there was no notable difference in 9 dimensions scores between no practice group and irregular practice group (all *P* > 0.05). Infants in regular practice group were higher in adaptability (*T* = 2.969, *P* = 0.004) and persistence (*T* = 2.493, *P* = 0.014), compared by infants in irregular practice group. Meanwhile, infants in regular practice group were lower in negative mood (*T* = 2.001, *P* = 0.047), while higher in adaptability (*T* = 5.009, *P* < 0.001), approach (*T* = 3.457, *P* = 0.001), and persistence (*T* = 3.259, *P* = 0.001), compared with infants in no practice group. As shown in Table 6, linear-regression analysis showed that higher adaptability, approachability, and persistence were still positively associated with the gentle regular tactile stimulation including other relative factors.

## 4. Discussion

The results of the current study demonstrate the effect of gentle tactile stimulation on the fetus in its temperament 3 months after birth. Significant difference in temperament type was found among infants in 3 groups at 3 months of age. In the regular practice group, the babies with easy type temperament accounted for 73.7%, which was higher than that in other two groups (*P* < 0.05). In addition, the infants who had received regular gentle tactile stimulation before birth had notably higher adaptability (*P* < 0.001), persistence (*P* = 0.003), and approach (*P* = 0.003), respectively. Infants in regularly practice group were lower in negative mood (*T* = 2.001, *P* = 0.047) while higher in adaptability (*T* = 5.009, *P* < 0.001), approach (*T* = 3.457, *P* = 0.001), and persistence (*T* = 3.259, *P* = 0.001), compared with infants in no practice group. The present data suggests that regular gentle tactile stimulation on fetus may promote the formation of easy type infant temperament.

Temperament type influences the relationship between the infant and the people around it, as well as the infant's physical and behavioral development [39]. A more easy-going temperament was associated with more well-being [40]. Aggressive children are likely to have more challenging temperament characteristics [41–44] that have the potential to influence the responses of others, setting up mutually reinforcing interpersonal interaction patterns that may maintain problem behavior over the long term [45]. Previous studies have found that infants with a difficult temperament remain irritable and are less likely to fit in with their environment for several years and may exhibit abnormal behavior in childhood [43, 46–48], while an easy temperament functions as a protective mechanism for these outcomes in social-emotional development [40, 46]. The results showed that the proportion of babies with easy temperament was notably higher in the regular practice group than in the irregular practice groups and no practice groups. The multivariable models showed that gentle tactile stimulation on the fetus was associated with an easy temperament although other variables which could affect temperament were included in the models.

Researches in disparate populations of children have consistently demonstrated an association of temperament characteristics such as approach/withdrawal, adaptability, persistence, and activity with externalizing behavior [43, 46–48]. The present study on infants showed that increased nocturnal sleep was correlated with increased approachability and increased diurnal sleep duration was also correlated with increased adaptability in 11-month-old baby [49]. Infants' early persistence and mothers' teaching are direct pathways to cognitive status at the start of the second year [50]. The study of Sakimura et al. indicates that 38.7% of 3- to 5-year-old children with aggressive behavior demonstrated low adaptability and low persistence [45]. Language development is also associated with adaptability and persistence [51]. The results showed that infants in regularly practice group were lower in negative mood and higher in adaptability, approach, and persistence, compared with infants in no practice group. Regular tactile stimulation can reduce mother's negative emotions and may calm the fetus directly. It can also attract fetus to cater to the stimulation of mothers and achieve higher approach and adaptability. Besides, if mothers insist to do regular tactile stimulation during pregnancy, fetus can also cultivate their perseverance to this interaction, and this kind of disposition may continue after birth. For these reasons, we concluded that the influence of gentle abdominal tactile stimulation on these dimensions may be good for the development of infant.

Nerve endings in skin are rich. Alberto Gallace's review showed that tactile sensations in interaction can have surprisingly powerful effects on people's behaviors and emotions. Specifically, touch appears to be capable of modulating people's compliance [52]. So we speculated one possible mechanism: the fetus lives in the warm amniotic fluid and tactile stimulation through the abdomen may enhance the flow of amniotic fluid and produces gentle stimulation on the fetal skin. This may satisfy the feeling of the fetus, which may be conducive to the formation of the easy type of infant temperament. On the other hand, when the pregnant woman gives tactile stimulation on the fetus, she may feel fetus' response more and her abdomen also receives more massage than often. These may relieve her anxiety and depression and reduce the level of cortisol in blood and amniotic fluid, which is believed to be good for the fetal psychological development and another possible mechanism [37]. However, considering that this is a retrospective research, further long-term studies are necessary to testify them.

Meanwhile there was no notable difference in the ratio of easy type between irregular practice group and no practice group, which indicated that irregular tactile stimulation has no significant effect on the formation of the easy type of infant temperament. Although the ratio of difficult type in irregular practice group was higher than other 2 groups, there was no significant difference among 3 groups. We speculated that there were two underlying reasons for this result: the sample size was not big enough; there was no relative effect indeed.

Although the present study illuminates that regular gentle tactile stimulation on fetus may promote the formation of easy type infant temperament, it is necessary to indicate some limitations of this study. Since this is retrospective design with potential for confounding of maternal behavior pre- and postnatally and maternal reporting of what happened in pregnancy may be influenced by what she is doing postnatally, then it is possible that mothers who massage their infants prenatally become attuned to their infant's cues and may be more sensitive. Besides, the roles of genetics, maternal personality, temperament, and mother's anxiety trait were not assessed at baseline when the intervention took place. However, it has been observed that maternal trait anxiety in the third trimester of pregnancy predicts infant difficult temperament when babies are four and six month old, independently of mother's antenatal and postnatal depression levels [30]. Therefore, the fact that in this study infants who received regular practice of tactile stimulation during pregnancy revealed more often easy temperament might be a result of mother's lower anxiety levels [53] already present during pregnancy, rather than the tactile stimulation intervention. Further studies will include this point to validate the findings of the study.

In summary, our study shows that regular gentle tactile stimulation on fetus may promote the formation of easy type infant temperament. Fetus undergoing regular gentle tactile stimulation show lower negative mood, improved notably higher approach/withdrawal, adaptability, and persistence. Further study and assessments should be done to deeply evaluate the regular gentle tactile stimulation on fetus in the formation of easy type infant temperament.

## Figures and Tables

**Table 1:** The comparison of demographic characteristics among the three groups.

Variables	Options	No practice group *N* (%)	Irregular practice group *N* (%)	Regular practice group *N* (%)	*P*
Maternal temperament tendency	Typical extrovert	68 (41.5)	28 (45.2)	16 (21.1)	0.002^a^
	Ambivert	86 (52.4)	34 (54.8)	58 (76.3)	
	Typical introvert	10 (6.1)	0	2 (2.6)	
	Total	164 (100)	62 (100)	76 (100)	

Maternal temperament stability	Typically stable	83 (50.6)	26 (41.9)	28 (36.8)	0.172^a^
	Ambivert	77 (47.0)	34 (54.8)	48 (63.2)	
	Typically unstable	4 (2.4)	2 (3.2)	0	
	Total	164 (100)	62 (100)	76 (100)	

Education	High school or below	12 (7.3)	2 (3.2)	0	0.529^a^
	College	106 (64.6)	48 (77.4)	64 (84.2)	
	Master	44 (26.8)	12 (19.4)	12 (15.8)	
	Doctor	2 (1.2)	0	0	
	Total	164 (100)	62 (100)	76 (100)	

Pregnant women as only child	No	50 (30.5)	9 (14.5%)	24 (31.6)	0.037^b^
	Yes	114 (69.5)	53 (85.5%)	52 (68.4)	
	Total	164 (100)	62 (100)	76 (100)	

Family annual income	≤5 000$	14 (8.6)	0	2 (2.6)	0.003^a^
	5 000–8 000$	18 (11.1)	6 (9.7)	8 (10.5)	
	8 000–16 000$	58 (35.8)	16 (25.8)	18 (23.7)	
	16 000–32 000$	40 (24.7)	20 (32.3)	26 (34.2)	
	≥32 000$	32 (19.8)	20 (32.3)	22 (28.9)	
	Total	162 (100)	62 (100)	76 (100)	

^a^Rank chi-square, ^b^Pearson chi-square.

The criteria of EPQA: ambivert, *T* score in E or N subscale is between 38.5 and 61.5; typical introvert: *T* score in E subscale <38.5; typical extrovert: *T* score in E subscale >61.5; typically nerve stable: *T* score in N subscale <38.5; typically nerve unstable: *T* score in N subscale >61.5.

**Table 2:** The comparison of maternal condition among the three groups.

Variables	Options	No practice group *N* (%)	Irregular practice group *N* (%)	Regular practice group *N* (%)	*P*
Working status	Full time job	43 (26.2)	14 (22.6)	18 (23.7)	0.790^a^
	Part time job	75 (45.7)	35 (56.5)	35 (46.1)	
	Unemployed	46 (28.0)	13 (21.0)	23 (30.3)	
	Total	164 (100)	62 (100)	76 (100)	

Musical fetal education	No	80 (48.8)	0	2 (2.6)	<0.001^d^
	Irregular practice	54 (32.9)	40 (64.5)	26 (34.2)	
	Regular practice	30 (18.3)	22 (35.5)	48 (63.2)	
	Total	164 (100)	62 (100)	76 (100)	

Talking with fetus	No	78 (47.6)	26 (41.9)	33 (44.6)	0.734^b^
	Yes	86 (52.4)	36 (58.1)	41 (55.4)	
	Total	164 (100)	62 (100)	74 (100)	

Delivery mode	Vaginal delivery	66 (40.2)	21 (33.9)	38 (50.0)	0.145^b^
	C-S	98 (59.8)	41 (66.1)	38 (50.0)	
	Total	164 (100)	62 (100)	76 (100)	

Chief caregiver	Mother	78 (47.6)	26 (41.9)	30 (39.5)	0.071^c^
	Father	80 (48.8)	32 (51.6)	46 (60.5)	
	Grand parents	2 (1.2)	4 (6.5)	0	
	Others	4 (2.4)	0	0	
	Total	164	62	76	

Mode of feeding	Pure breast feeding	40 (24.4)	14 (22.6)	15 (19.7)	0.820^b^
	Mixed feeding	100 (70.0)	41 (66.1)	52 (68.4)	
	Artificial feeding	24 (14.6)	7 (11.3)	9 (11.8)	
	Total	164 (100)	62 (100)	76 (100)	

Depression or anxiety	No	134 (81.7)	49 (79.0)	64 (84.2)	0.735^b^
	Yes	30 (18.3)	13 (21)	12 (15.7)	
	Total	164 (100)	62 (100)	76 (100)	

^a^Rank chi-square, ^b^Pearson chi-square, ^c^Fisher's exact, and ^d^rank correlation test.

Norm of SDS in China: score ≥ 53 as “depression.”

Norm of SAS in China: score ≥ 50 as “anxiety.”

**Table 3:** Comparison of infant temperament type among the three groups *N* (%).

Temperament type	Options	Groups			*P*
		No practice group	Irregular practice group	Regular practice group	
Different types	Easy	69 (42.1)	33 (53.2)	56 (73.7)	<0.001^a^
	Intermediate	85 (51.8)	23 (37.1)	17 (22.4)	
	Difficult	6 (3.7)	4 (6.5)	1 (1.3)	
	Slow	4 (2.4)	2 (3.2)	2 (2.6)	
	Total	164 (100)	62 (100)	76 (100)	

Easy type	Easy	69 (42.1)	33 (53.2)	56 (73.7)	<0.001^b^
	Others	95 (57.9)	29 (46.8)	20 (26.3)	
	Total	164 (100)	62 (100)	76 (100)	

Difficult type	Difficult	6 (3.7)	4 (6.5)	1 (1.3)	0.289^a^
	Others	158 (96.3)	58 (93.5)	75 (98.7)	
	Total	164 (100)	62 (100)	76 (100)	

^a^Fisher's exact; ^b^Pearson chi-square.

The temperament type is defined by rhythmicity, approach/withdraw, adaptability, intensity, and mood dimensions. Easy type: among 5-dimension scores, not more than 2 is above the average and none is more than 1 standard deviation. Difficult type: 4- or 5-dimension scores are above the average, including intensity dimension, and 2-dimension scores are more than 1 standard deviation. Slow type: it is similar to difficult type, but approach/withdraw or adaptation dimension score is more than 1 standard deviation. Meanwhile, activity dimension score is not more than 1/2 standard deviation and mood dimension score is not less than 1/2 standard deviation. Intermediate type: all others.

**Table 4:** The results of logistic multivariable analysis of infant temperament type on independent factors (*N* = 300^b^).

	OR^c^	95.0% C.I.		*P*	OR^a^	95.0% CI		*P*
		Lower	Upper			Lower	Upper	
Gentle tactile stimulation (none)	Ref (dnm *b* variable)							

Gentle tactile stimulation (irregular)	1.567	.871	2.819	0.134	1.598	.785	3.251	0.196

Gentle tactile stimulation (regular)	3.855	2.121	7.006	<0.001	5.098	2.457	10.579	<0.001

Pregnant woman being only child					1.283	1.019	1.615	0.034

Annual family income					.652	.365	1.165	0.149

Temperament tendency of woman					.331	.205	.532	0.000

Musical fetal education (none)	Ref (dnm *b* variable)							

Musical fetal education (irregular)					.805	.438	1.479	0.484

Musical fetal education (regular)					1.170	.541	2.533	0.689

^a^Adjusted OR (adjusted for confounding factors: musical fetal education, temperament tendency of woman, pregnant woman being only child, and annual family income).

^c^Crude OR.

^b^2 cases which did not answer the question about family income were excluded.

**Table 5:** The comparison of nine dimensions of infant temperament among the three groups (*N* = 302).

	Groups							
Dimensions	No practice group		Irregular practice group		Regular practice group		*F*	*P* ^a^
	M	SD	M	SD	M	SD		
Activity	3.342	0.449	3.234	0.385	3.229	0.33	2.736	0.066
Rhythmicity	3.311	0.706	3.234	0.666	3.273	0.72	0.288	0.750
Adaptability	2.995	0.627	2.882	0.618	2.56	0.646	12.408	<0.001
Approach/withdrawal	2.377	0.507	2.243	0.48	2.14	0.516	6.064	0.003
Intensity	3.334	0.547	3.38	0.428	3.297	0.519	0.251	0.778
Mood	2.725	0.481	2.716	0.52	2.588	0.56	1.989	0.139
Persistence	2.742	0.594	2.706	0.487	2.463	0.657	5.984	0.003
Distractibility	2.352	0.663	2.428	0.521	2.295	0.629	0.735	0.481
Threshold	4.447	0.67	4.412	0.719	4.31	0.707	1.04	0.355

^a^ANOVA test (the higher dimension score indicates higher activity, weaker rhythmicity, lower approach, weaker adaptability, stronger intensity, negative mood, shorter persistence, higher distractibility, and lower threshold.).

**Table 6:** Multivariable linear-regression analysis of the score of 3 dimensions of the babies on independent factors (*N* = 300^b^).

Dependent variable	Self-variable	Crude estimated *B*	95.0% CI		*P*	Adjusted estimated *B* ^a^	95.0% CI		*P*
			Lower Bound	Upper Bound			Lower Bound	Upper Bound	
Adaptability	Gentle tactile stimulation (irregular)	−.127	−.314	.061	0.185	−0.084	−.294	.126	0.432
	Gentle tactile stimulation (regular)	−.449	−.623	−.274	0.000	−0.427	−.628	−.226	0.000

Approach/withdrawal	Gentle tactile stimulation (irregular)	−.142	−.291	.006	0.059	−.154	−.323	0.014	0.072
	Gentle tactile stimulation (regular)	−.245	−.383	−.107	0.001	−.259	−.420	−.097	0.014

Persistence	Gentle tactile stimulation (irregular)	−.035	−.208	.138	0.693	.026	−.174	0.225	0.801
	Gentle tactile stimulation (regular)	−.278	−.439	−.117	0.001	−.226	−.417	−.035	0.020

^a^Adjusted for confounding factors: musical fetal education, temperament tendency of woman, pregnant woman being only child, and annual family income.

^b^2 cases which did not answer the question about family income were excluded.
